# Solubility Enhancement of Ibuprofen by Adsorption onto Spherical Porous Calcium Silicate

**DOI:** 10.3390/pharmaceutics13060767

**Published:** 2021-05-21

**Authors:** Yayoi Kawano, Shiyang Chen, Takehisa Hanawa

**Affiliations:** Faculty of Pharmaceutical Sciences, Tokyo University of Science, 2641, Yamazaki, Noda 278-8510, Chiba, Japan; y.kawano@rs.tus.ac.jp (Y.K.); csy1991710@gmail.com (S.C.)

**Keywords:** spherical calcium silicate, sublimation, ibuprofen, sealed heating, amorphous, evaporation

## Abstract

The solubility of a drug is higher when it is in an amorphous form than when it is in a crystalline form. To enhance the solubility of ibuprofen (IBU), a poorly water-soluble drug, we attempted to adsorb IBU onto spherical porous calcium silicate (Florite^®^ PS300, PS300) in two ways: the evaporation (EV) and sealed heating (SH) methods. The crystallinity of the samples was evaluated using powder X-ray diffraction analysis (PXRD) and differential scanning calorimetry (DSC). The molecular interaction between IBU and PS300 was evaluated with FTIR. In addition, the dissolution behavior of IBU in the samples was assessed by the dissolution test. Based on the results of the PXRD and DSC measurements, both methods allowed adsorption of IBU onto PS300, and IBU was amorphized. Based on the FTIR observations, in the SH or EV mixtures containing 10% and 30% IBU, respectively, it seemed that the IBU molecules intermolecularly interacted with calcium molecules as the main component of PS300. Improvement in the solubility of IBU was observed with both methods; however, the dissolution rate of IBU from samples prepared via SH was higher than that from EV, or of IBU crystals. Collectively, our findings indicate that the petal-like structure of PS300, which has a spherical shape and good flowability, is an effective tool for adsorbing IBU onto PS300 via SH.

## 1. Introduction

Poorly water-soluble drugs make up a high percentage of currently marketed products. In addition, more than 40% of oral drug products in pharmaceutical markets contain poorly water-soluble drugs [1,2,3]. Recently, as the number of drug candidates in drug discovery has increased, in parallel, poorly water-soluble drugs have also steadily increased; almost 70% of new drug candidates have poor water solubility [4]. These drugs are orally administered. Therefore, it is essential to improve their bioavailability (BA). One of the indexes to classify BA, the Biopharmaceutics Classification System (BCS), is defined by solubility and permeability and includes Classes I to IV. In formulation studies, in Class II drugs, the dissolution rate limits absorption; therefore, solubility enhancement is an important issue for developing oral dosage forms. Many approaches to improve the solubility of poorly water-soluble drugs, such as amorphization, nanoparticles [5,6,7], co-crystals [8,9,10], liposomes [11,12,13], microemulsions [14,15,16], self-emulsifying drug delivery systems [17,18,19,20,21], and drug-cyclodextrin inclusion compounds [22,23], have been reported. Amorphization, which includes spray drying, freeze-drying, and grinding with a water-soluble polymer using a ball mill, is one of the most popular and simple methods of improving solubility [24,25,26,27,28,29]. There are, however, both advantages and disadvantages regarding their physicochemical properties. The development of liposomes, microemulsions, and self-emulsifying systems is relatively easy; however, as these are liquid formulations, their stability often becomes an issue. Solid dispersions and nanocrystalline formulations require special techniques for manufacturing. Additionally, their physicochemical stability is considered to be insufficient. 

Adsorption to porous materials is another amorphization method in which crystal forms change to an amorphous state. Yonemochi et al. reported the amorphization of drugs using controlled porous glass [30]. In addition, Yuasa et al. reported a method for absorbing oily vitamin E into porous calcium silicate [31]. In addition, for the improvement in the solubility of poorly water-soluble drugs, using adsorption with organic solvents on porous calcium silicate [32,33,34,35] and the formation of solid dispersion with porous calcium silicate using a spray drying method [32] have been reported. Many organic solvents are typically used for dissolving drugs in lots of these methods. 

Florite^®^ (FLR) is a porous calcium silicate with a petal-like structure [36]. The pore size has a distribution with two peaks at 12 and 0.15 μm, which can be attributed to interparticle and intraparticle pores, respectively [31]. The pores show excellent water and oil absorption into intra-particles, alongside their formation ability. The absorption of drugs into porous carriers by solvent elimination and grinding methods [35,36] has also been reported. The use of organic solvents does, however, cause several problems, including environmental pollution and toxicity due to residual solvents [37].

On the other hand, the grinding of a mixture of drugs and polymers does not use organic solvents. Therefore, the adsorption methods using porous carriers without organic solvents can be a solution to the above problems. In a previous study, we reported the adsorption of a poorly water-soluble drug into an FLR by the sealed heating method, which does not use organic solvents [38]. Problems can occur if used for oral dosage tablets because FLR is difficult to handle due to its low flowability and high dustiness. Some recent studies have reported that granules including FLR, and poorly water-soluble drugs can be prepared by the wet granulation method to improve the low flowability [39,40,41]. Florite^®^ PS300 (PS300) was developed in response to these problems, and its shape is spherical. Therefore, here we focused on the PS300 of FLR as a porous carrier, which is granulated from FLR and has high flowability and compressibility [38]. 

Ibuprofen (IBU) was used as a model for poorly water-soluble drugs in this study. IBU is an example of a poorly water-soluble drug from the Class II drugs of the BCS, with low solubility and high permeability. In addition, IBU, with a melting point of 75–77 °C, exhibits sublimation. The adsorption of IBU onto PS300 via evaporation (EV) and sealed heating (SH) was investigated. As mentioned above, EV is a popular method for the adsorption of drugs dissolved by organic solvents.

On the other hand, SH can adapt to the adsorption of sublimable drugs into porous carriers. In the present study, PS300 was examined for feasibility as a drug carrier, with the aim of improving the solubility of poorly water-soluble drugs. The EV and SH methods were used to try to obtain an amorphous state of IBU with PS300. In addition, nonporous calcium silicate (CaSiO_3_) and zirconia beads were used as reference materials, and the effects of the presence or absence of porous features on the surface were compared. In this study, the physicochemical properties of various samples were investigated using differential scanning calorimetry (DSC), powder X-ray diffractometry (PXRD), Fourier-transform infrared (FTIR) spectroscopy, and the dissolution test to evaluate the feasibility of using PS300 as a carrier for improving the solubility of poorly water-soluble drugs.

## 2. Materials and Methods

### 2.1. Chemical Reagents

PS300 was generously provided by Tomita Pharmaceutical Co., Ltd. (Tokushima, Japan). CaSiO_3_ was purchased from Wako Pure Chemical Industries, Ltd. (Osaka, Japan). IBU was generously provided by Kaken Pharmaceutical Co., Ltd. (Tokyo, Japan). Zirconia beads of 0.3 mm in diameter were purchased from Nikkato Co., Ltd. (Osaka, Japan). Ethanol, 1 mol/L sodium hydroxide solution, calcium chloride, phosphoric acid, acetonitrile, and distilled water were purchased from Kanto Chemical Co., Inc. (Tokyo, Japan). All received chemicals and solvents were of analytical reagent grade.

### 2.2. Preparation of Samples

#### 2.2.1. Preparation of Physical Mixtures

The PS300 and IBU were sieved with a 36-mesh (425 μm) sieve; then, physical mixtures (PMs) of various mixing ratios were obtained by mixing them in a glass vial using a VORTEX^®^ mixer (Table 1). 

#### 2.2.2. Adsorption of IBU onto Drug Carriers

##### EV Method

The IBU was dissolved in ethanol and poured, along with PS300, into an eggplant flask. Then, the IBU was adsorbed onto the PS300 by evaporating and removing the ethanol with a rotary evaporator. The resulting white powder was an evaporated mixture (EVM). The EVM was dried further in a vacuum desiccator at 25 °C for 6 h. It was then stored in a desiccator under a definite relative humidity (RH 22%) until further use.

##### SH Method

Various PMs were placed into 20 mL vacuum reaction tubes (ThermoFisher Scientific Inc., Waltham, MA, USA) and heated under reduced pressure (−96 kPa) using a vacuum pump for 6 h. The sealed and heated mixture (SHM) was obtained by heating at 70 °C. The obtained SHM was then stored in a desiccator under a definite relative humidity (RH 22%) until further use.

#### 2.2.3. Preparation of IBU Calcium

IBU (10.3 g) was dissolved in a 1 mol/L sodium hydroxide solution (50 mL), then calcium chloride (2.78 g) was added, and the solution was mixed for 1 h. The mixture was filtered through a 0.45 μm membrane filter and dried in a desiccator at 30 °C for 24 h.

### 2.3. Physicochemical Properties of Various Samples

The surface observation of PS300 and CaSiO_3_ was carried out using a scanning electron microscope (SEM, JSM-6390LA, JEOL Ltd., Tokyo, Japan). 

The particle diameter of PS300 and CaSiO_3_ was measured by a Multisizer 4e (Beckman Coulter, Inc., Brea, CA, USA) under the following conditions: 2-propanol containing 3% ammonium thiocyanate was used as the dispersion media, the aperture sizes were 200 μm, 400 μm, and 2000 μm, and the measurement ranges were 4 to 120 μm, 2.8 to 84 μm, and 40 to 1200 μm.

The flowability of PS300 and CaSiO_3_ was evaluated by the repose angle, performed by the injection method, using a repose angle measurement instrument (AOR-057, Tsutsui Scientific Instrument Co., Ltd., Tokyo, Japan).

The density of PS300 and CaSiO_3_ was measured by a gas pycnometer (AccuPyc II 1340TC-10CC, Micromeritics Corporation Headquarters, Norcross, GA, USA) under the following conditions: the displacement medium was helium gas, the setup and operation pressure was 134.45 kPa; the counts of purge and operation were 10 times each; the equilibrium pressure was 0.034 kPa/min, and the measurement temperature was 23 °C.

The specific surface area and pore volume of PS300 and CaSiO_3_ were measured by the gas adsorption method under nitrogen gas and were calculated by the Brunauer–Emmett–Teller method, using the micromeritics automatic surface area and a porosimetry analyzer (TriStar II 3020, Micromeritics Corporation Headquarters, GA, USA). As a pretreatment, a vacuum evacuating device (VacPrep 061, Micromeritics Corporation Headquarters, GA, USA) was used at 150 °C for 150 min.

### 2.4. Powder X-ray Diffractometry (PXRD)

PXRD was used to evaluate the crystallinity of the samples. The patterns were obtained from a RINT 2000 diffractometer (Rigaku Co., Tokyo, Japan) a cumulative number of four times, under the following conditions: target Cu with a Ni filter, a voltage of 30 kV, a current of 5 mA, scanning speed 5°/min, and a diffraction angle range of 5 to 40°.

### 2.5. Differential Scanning Calorimetry (DSC)

Thermal analysis of the samples was performed using a DSC instrument (DSC-60plus, Shimadzu Co., Ltd., Kyoto, Japan) under nitrogen gas flow and measured at a heating rate of 10 °C/min. Each sample was heated at a temperature range of 25–110 °C. Each mixture, including 2 mg IBU, was used for the measurement. The percentage of the crystalline region of the IBU was calculated using Equation (1), as described by Pan et al. [42,43,44].

Percentage of crystalline region of IBU (%) = ΔH_PM_/(ΔH_IBU_ × Conc_IBU_(%)) × 100 (1)

ΔH_PM_ and ΔH_IBU_ show the enthalpy of fusion (J/g) of IBU in various PMs and IBU crystals, respectively, and Conc (%) leads the IBU concentration in PMs.

In addition, the percentage of the amorphized region of the IBU was calculated by Equation (2).

Percentage of the amorphized region of IBU (%) = 100 − Percentage of the crystalline region of IBU (%)(2)

### 2.6. Fourier-Transform Infrared Spectroscopy (FTIR)

Evaluation of the molecular states between each sample was performed by a Frontier FT-IR Spectrometer (PerkinElmer Inc., Waltham, MA, USA). The thickness of the sample was 1.0 mm, and the scanning range was 4000 to 500 cm^−1^. The spectra were taken using a single bounce diamond anvil attenuated total reflection (ATR) cell. In addition, IR spectra with temperature rises were measured using high-performance ATR (Cladi ATR^TM^, PIKE Technologies, Madison, WI, USA).

### 2.7. Dissolution Study

The dissolution behaviors of various samples were assessed with the JP, 17th edition paddle method, at 37.0 ± 0.5 °C, paddle rotation speeds of 100 rpm, and test fluid of 500 mL of distilled water. Various mixtures containing 50 mg of IBU were poured into the test fluid. A definite volume (5 mL) of sample solutions was removed and immediately replaced with 5 mL of distilled water at appropriate time intervals. The collected sample solution was filtered through a 0.45 μm membrane. The concentration of IBU was measured by HPLC (LC-20AD, Shimadzu Co., Ltd., Kyoto, Japan, λ_max_; 214 nm, flow rate; 1.0 mL/min, mobile phase; phosphate buffer (pH 2.0): acetonitrile = 46:54 (*v*/*v*), measurement temperature; 30 °C). Samples of 5 μL volume were injected onto a C18 column (Shodex^®^ (C18M 4D), 4.6 mm × 150 mm, Showa Denko K.K., Tokyo, Japan). The percentage of released IBU was calculated based on the theoretical value of IBU, along with the averages of three determinations.

## 3. Results and Discussion

### 3.1. Physicochemical Properties of Various Samples

**Figure 1:**
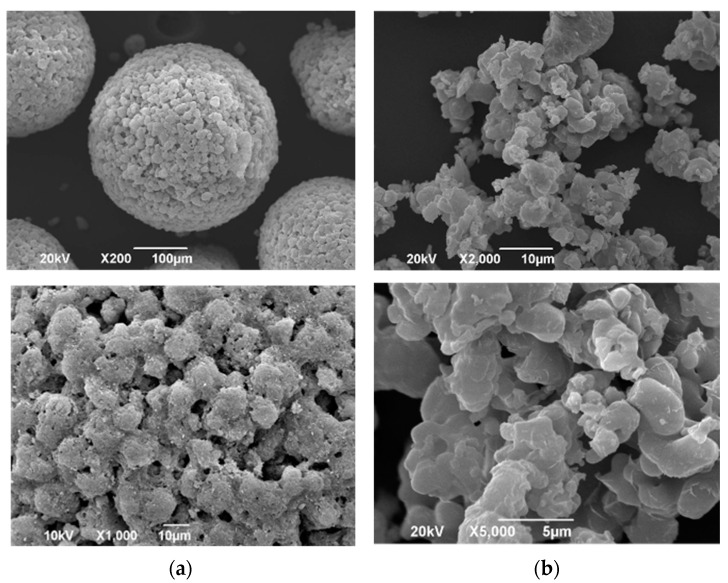
SEM images of PS300 and CaSiO_3_. (**a**) PS300; (**b**) CaSiO_3_.

SEM images of PS300 and CaSiO_3_ are shown in Figure 1. In the image of the PS300, the particles exhibit a spherical shape and a unique petal-like structure with a markedly porous nature (Figure 1a). The surface of the CaSiO_3_ is smooth, and no porous nature is observed (Figure 1b).

The various characteristics of PS300 and CaSiO_3_ are summarized in Table 2. The average diameter of PS300 was 280.2 μm, which is about 20-fold larger than that of CaSiO_3_. The angles of repose of PS300 and CaSiO_3_ were 18.0° and 47.9°, respectively. Therefore, PS300 had high flowability. In addition, their densities were almost identical. The surface area and pore volume of PS300 and CaSiO_3_ were 177.2 m^2^/g and 1.33 m^2^/g, and 0.723 cm^3^/g and 0.003 cm^3^/g, respectively. The PS300 was 270-fold and 241-fold larger than the CaSiO_3_. In our previous study, we reported that the specific surface area and total pore volume of FLR were 157.9 m^2^/g and 0.656 cm^3^/g, respectively. FLR was shown to be useful as a porous carrier for the SH method to adsorb sublimable drugs [38]. The specific surface area of the PS300 was larger than that of the FLR. In addition, the total pore volume was also bigger than that of the FLR. Furthermore, the angle of repose of PS300 was about half that of the FLR (data not shown). Due to its good flowability, PS300 seems to apply to the formulation additives. 

### 3.2. Powder X-ray Diffractometry (PXRD)

**Figure 2:**
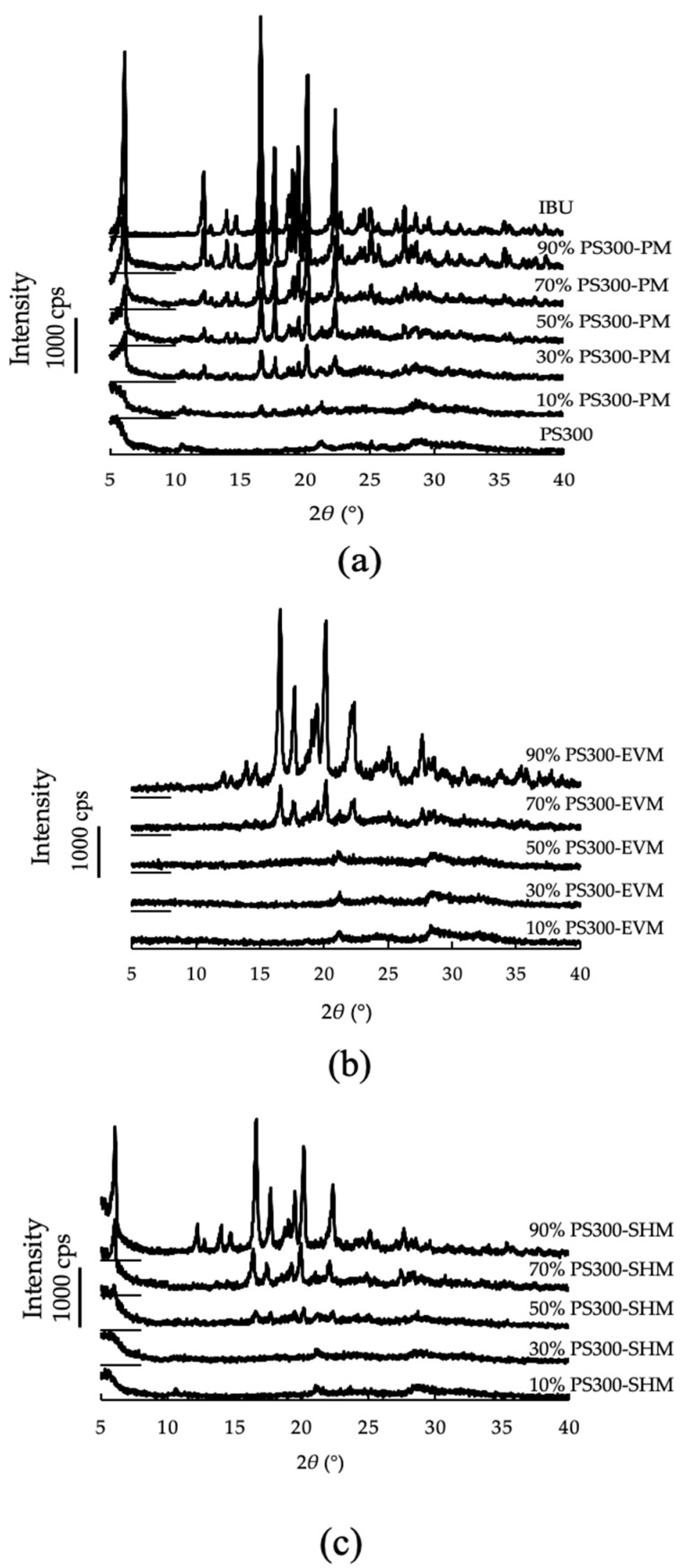
PXRD patterns of various samples. (**a**) IBU, PS300, and PMs of various mixing ratios; (**b**) EVMs of various mixing ratios; (**c**) SHMs of various mixing ratios.

Figure 2a shows the PXRD patterns of the PS300, CaSiO_3_, IBU crystals, and various PMs. In the diffraction pattern of the IBU crystals, proper peaks at 2*θ* = 6.1°, 12.2°, 16.6°, 20.18°, and 22.3° were observed [44]. On the other hand, the diffraction pattern of the PS300 showed diffraction peaks due to CaSiO_3_ at 2*θ* = 10.5°, 20.8°, and 28.1° [45]. In the diffraction pattern of the PMs, all samples showed prominent diffraction peaks due to IBU, although the intensity became lower as the mixing ratio of IBU decreased.

Figure 2b shows the PXRD patterns of samples obtained by the EV method (PS300-EVM). The diffraction patterns of PS300-EVM, containing up to 70% IBU, had diffraction peaks due to the IBU crystals at 2*θ* = 16.6° and 20.2°. Those peaks disappeared in PS300-EVM containing 50% or less IBU.

Figure 2c shows the PXRD patterns of samples obtained by the SH method (PS300-SHM). The diffraction patterns of PS300-SHM, containing up to 50% IBU, had diffraction peaks due to the IBU at 2*θ* = 16.6° and 20.2°. The peaks due to IBU crystals disappeared in the diffraction patterns of PS300-SHM containing 30% or less IBU.

### 3.3. Differential Scanning Calorimetry (DSC)

**Figure 3:**
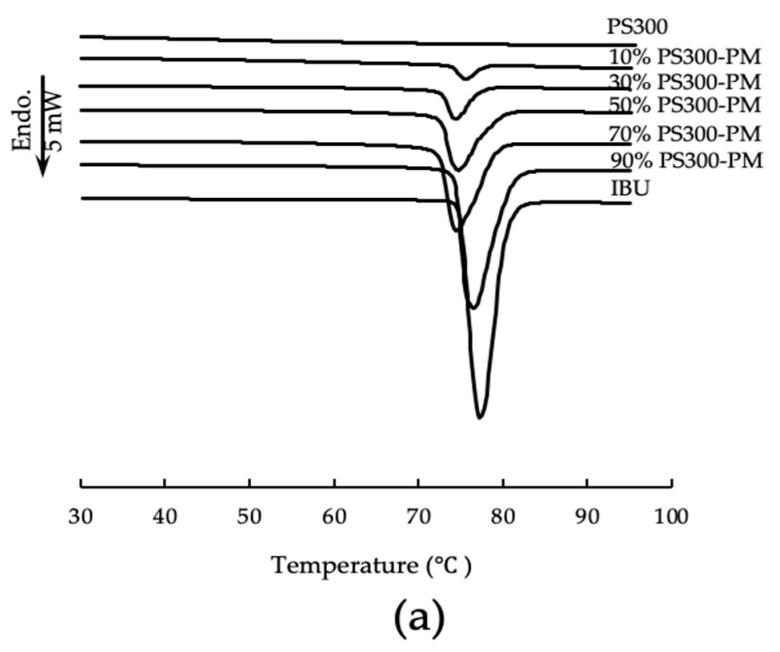

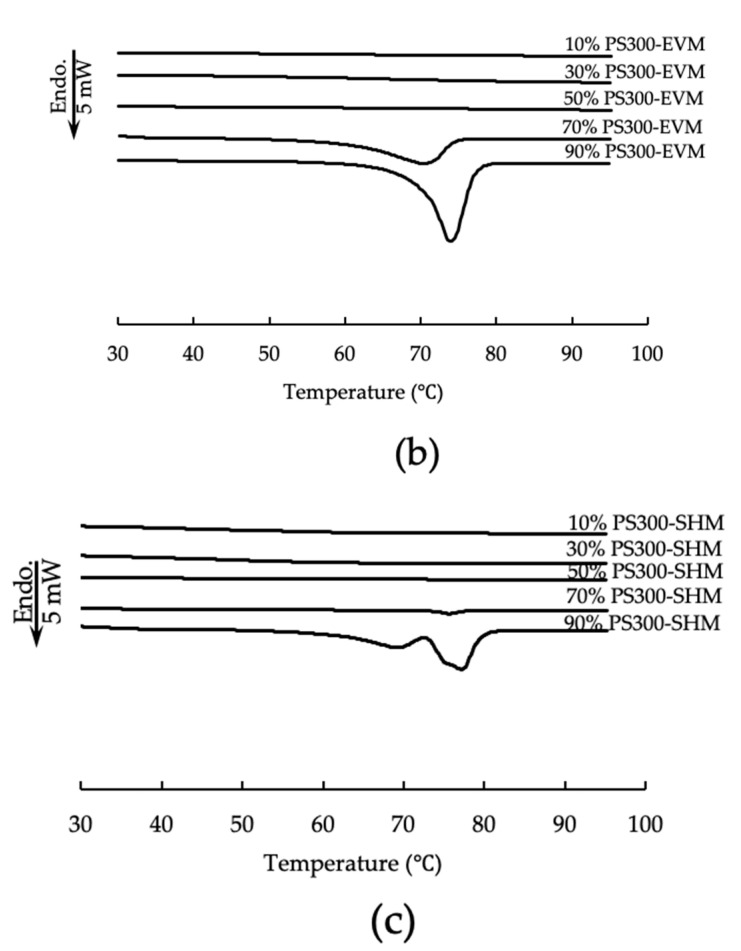
DSC curves of various samples. (**a**) IBU, PS300, and PMs of various mixing ratios; (**b**) EVMs of various mixing ratios; (**c**) SHMs of various mixing ratios.

The DSC curves of the PS300, IBU crystals, and various PMs are shown in Figure 3a. Neither endo- nor exothermic peak due to PS300 was observed in the DSC curves; however, endothermic peaks due to the melting of IBU crystals were observed at 74–83 °C on the DSC curves of all PMs.

Figure 3b,c shows the DSC curves of samples obtained by PS300-EVM and PS300-SHM, respectively. Endothermic peaks due to the melting of IBU crystals were observed up to a content of 70% IBU in PS300-EVM and PS300-SHM; however, endothermic peaks disappeared at 30% or less IBU content in PS300-EVM and PS300-SHM. In addition, two endothermic peaks at 90% IBU were observed in PS300-SHM. Nakai et al. reported that two endothermic peaks of benzoic acid, which were adsorbed onto porous carriers, appeared in their DSC curves [45,46]. In their study, benzoic acid was in the carriers as crystals, disordered structures, and in an amorphous state. The endothermic peaks, due to the melting point of the disordered system, were lower than those of the crystals. We believe that the same was true for our experiment. In other words, it was considered that there were three phases of IBU molecules in the PS300 mixture, namely, IBU crystals, disordered IBU crystals, and the amorphous state, in the porous nature of the PS300 [46,47]. In our previous study, this phenomenon was not observed, and FLR does not have a spherical shape as a carrier. FLR has a porosity that is deep and large, with a petal structure. From the results of mercury porosimetry, the pore size of FLR was larger than that of PS300, with an average of 173.7 nm and 139.6 nm, respectively (data not shown). Nakai et al. also reported that the pore size of the porous carrier affects the molecular state when adsorbing drugs. Therefore, three molecular states were also suggested for PS300 in this study.

**Figure 4:**
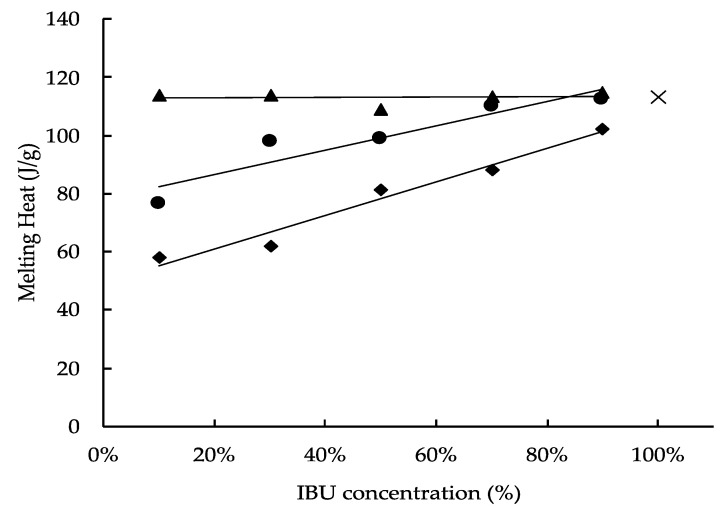
The enthalpy of fusion of IBU in various samples. ×: IBU crystals; ◆: PS300-PMs; ●: CaSiO_3_-PMs; ▲: ZrO_2_ Beads-PMs.

**Figure 5:**
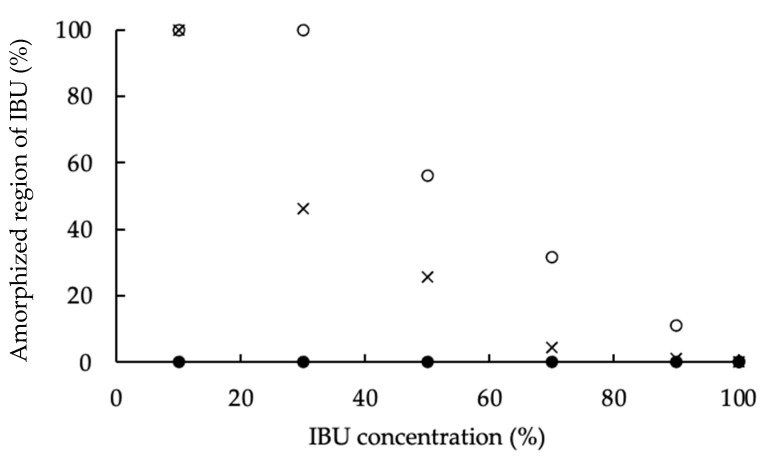
Percentage of the amorphized region of IBU in various samples. ○: PS300-PMs; ×: CaSiO_3_-PMs; ●: ZrO_2_ Beads-PMs.

Osawa et al. reported that the changes in the percentage of the amorphous state of a drug concentration in a solid dispersion could be calculated from DSC curves [43]. Therefore, in this study, to conjecture the crystallinity of IBU in the PMs of PS300, CaSiO_3_, which does not have the visually marked porous nature of PS300, and zirconia beads, which do not have a porous character either and have high sphericity, were used as control carriers and were evaluated by DSC measurement. The enthalpy of fusion (J/g) of IBU in various PMs and IBU crystals were shown in Figure 4. The endothermic peak was then derived from the melting of the IBU crystals in various samples, and the percentage of the crystalline region of IBU (%) was calculated by Equation (1). At the same time, the percentage of the amorphized region of IBU was calculated by Equation (2) (Figure 5).

The melting heat of IBU in the PMs prepared with zirconia beads showed no clear difference from the melting heat of IBU crystals. Therefore, no interaction was seen between IBU molecules and the surface of the zirconia beads, which do not have a porous structure. On the other hand, the melting heat of IBU in the PMs prepared with CaSiO_3_ and PS300 showed lower values with a decrease in the IBU content. In the PMs of the same weight ratio, the melting heat of IBU with CaSiO_3_ was higher than with PS300. In other words, from the results of the DSC measurements, it was confirmed that the amorphization of IBU was greater than that of CaSiO_3_ because both the surface area and the pore volume of PS300 were larger than those of CaSiO_3_. Additionally, in this study, when the adsorbed amount of IBU was about 30% of the PS300 weight, it was deemed that all IBU molecules were reachable in the deep portion of the PS300 pores as a gas phase (Figure 5).

### 3.4. Fourier-Transform Infrared Spectroscopy (FTIR)

**Figure 6:**
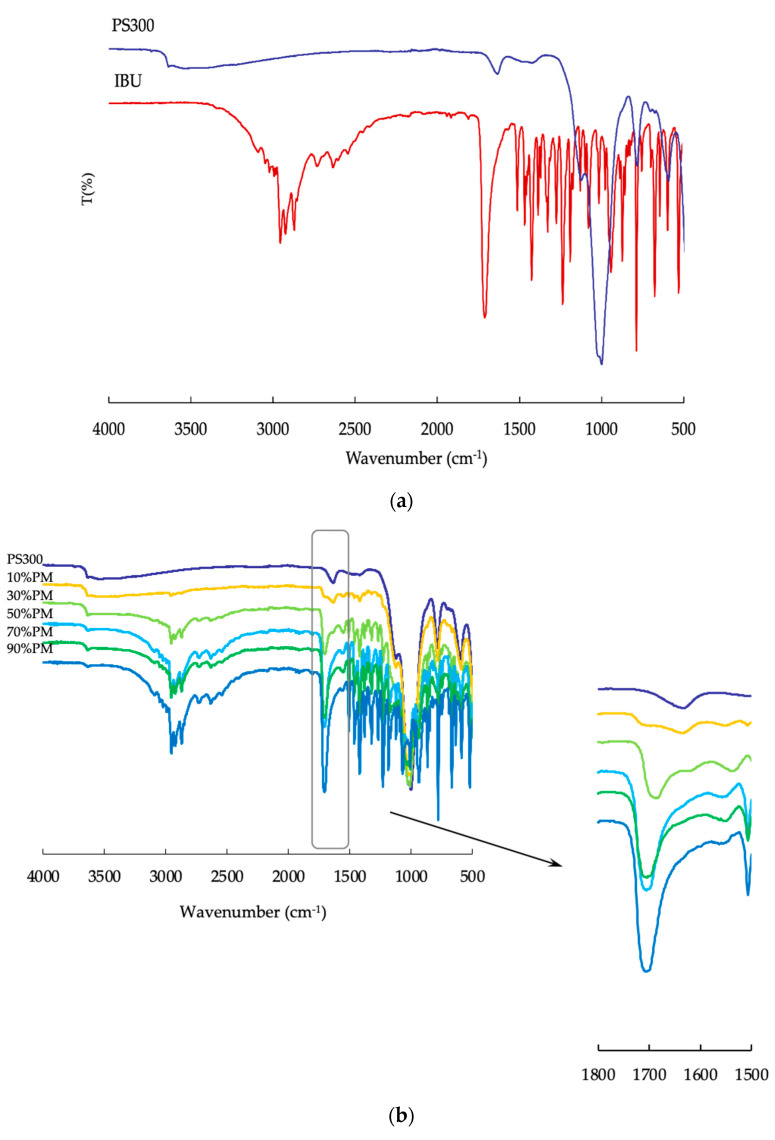

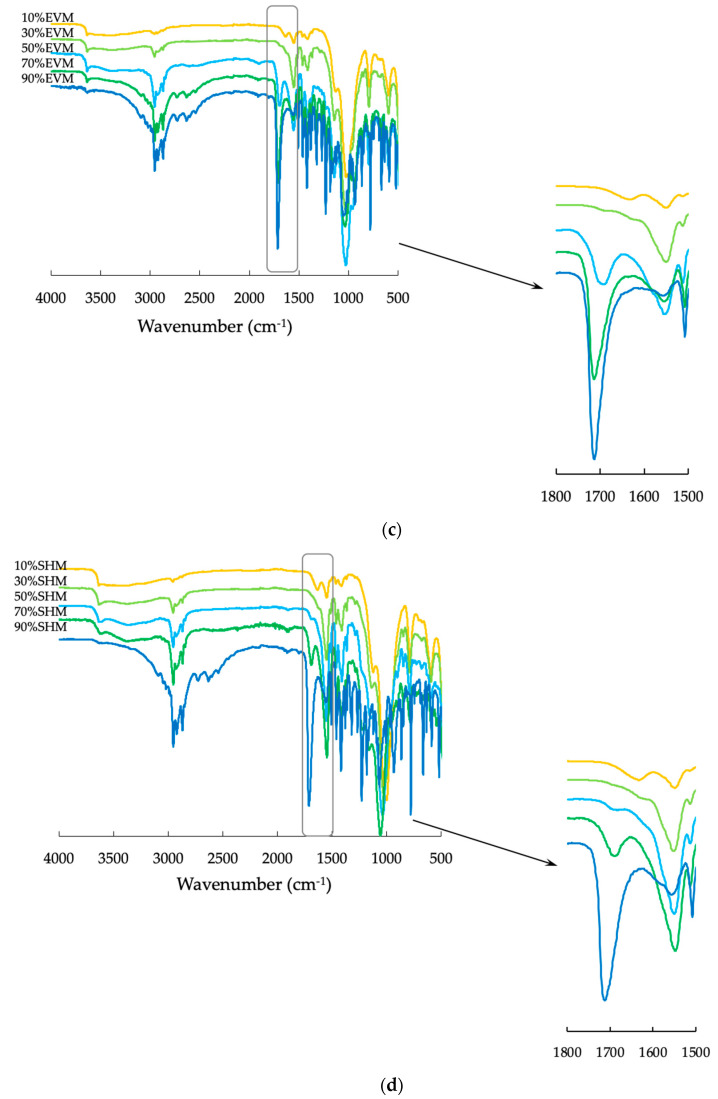
FTIR spectra of various samples. (**a**) PS300, and IBU crystals; (**b**) PMs; (**c**) EVMs; (**d**) SHMs.

We evaluated the molecular interactions between the surface of the PS300 and IBU molecules using ATR-FTIR. On the IR spectrum of the IBU, a prominent peak of the C=O stretching vibration band at 1710 cm^−1^ was observed, as shown in Figure 6a. In addition, in the IR spectrum of PS300, the peak was due to the -OH bending vibration band at 1635 cm^−1^, as shown in Figure 6a. All samples had a peak in the PMs due to the C=O stretching vibration band at 1710 cm^−1^; they also had a peak at 1550 cm^−1^, as shown in Figure 6b. In EVMs and SHMs, the peak at 1710 cm^−1^ disappeared when the content of IBU was 10% and 30% in the mixtures, and the peak intensity of 1550 cm^−1^ was stronger than at other contents of IBU, as seen in Figure 6c,d. As reported in our previous study, adsorbed IBU forms calcium salt due to the interaction between the IBU and calcium on the surface of the PS300 [37]. In this study, it was confirmed that calcium salt was formed on the surface during the adsorption of IBU onto PS300 (data not shown).

**Figure 7:**
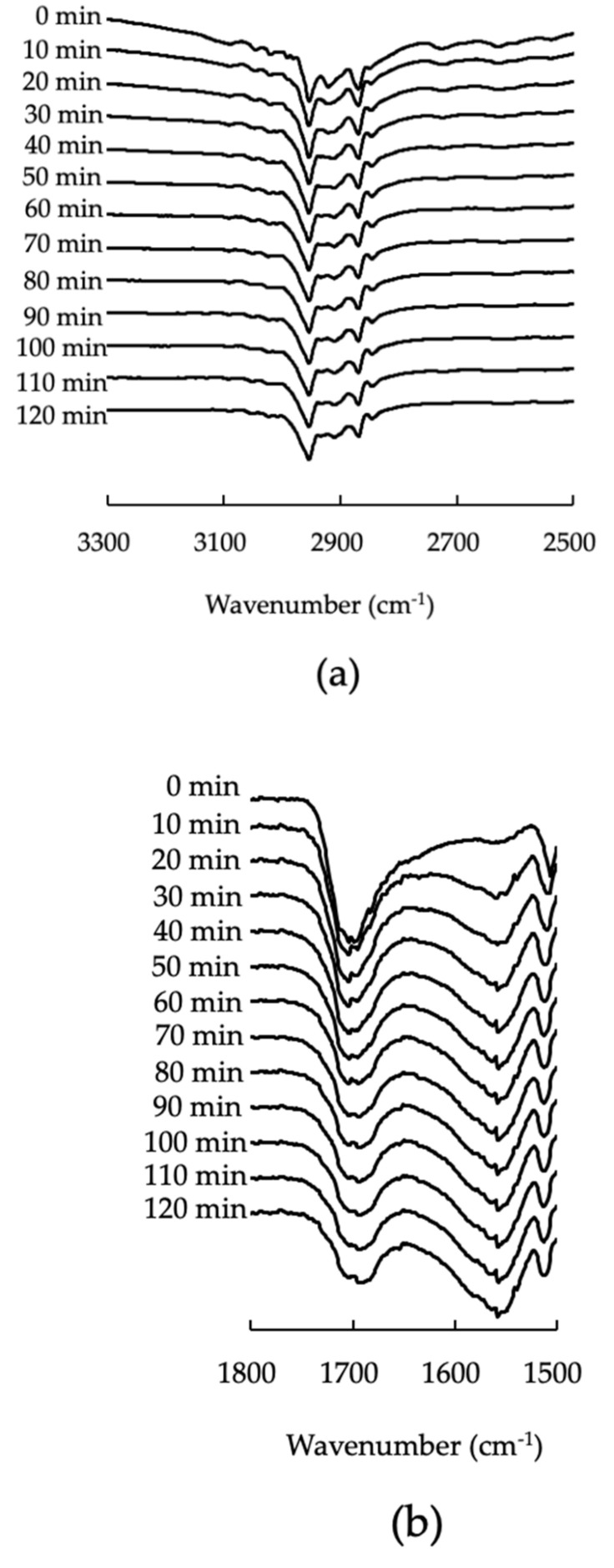
FTIR spectra of 50% PS300-PM, at different time points (0–120 min), at 70 °C. (**a**) From 3300–2500 cm^−1^; (**b**) from 1800–1500 cm^−1^.

As mentioned above, calcium salt of IBU was formed by interactions between IBU and calcium on the surface of PS300. ATR spectra were measured over time to clarify the changes in the IBU’s molecular state during heating. Figure 7 shows the FTIR spectra at 3300–2500 cm^−1^ (OH) and 1800–1500 cm^−1^ (C=O), of 50% PS300-PM, at different time points, at 70 °C.

The peak intensity of 1710 cm^−1^, and that due to the -OH of 3300–2500 cm^−1^, decreased within 90 min of heating. The peak intensity of 1550 cm^−1^ increased; however, the peak intensity did not change from 100 min after measurement. This indicates that, in a 50% PS300-PM system, IBU calcium was formed just after heating.

### 3.5. Dissolution Study

**Figure 8:**
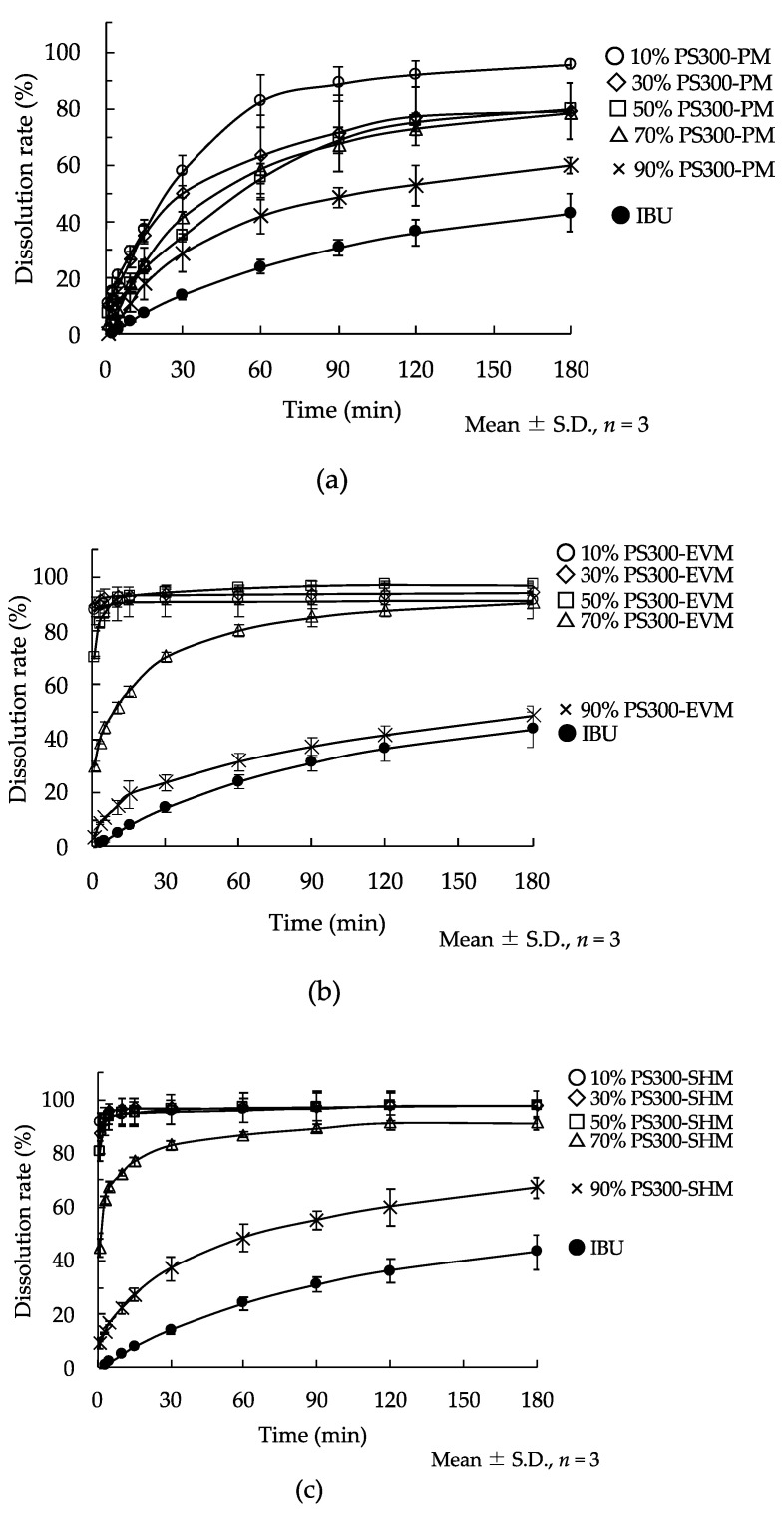
Dissolution behavior of IBU from IBU crystals and various samples. (**a**) PM: ●: IBU crystals; ○: 10% PS300-PM; ◇: 30% PS300-PM; □: 50% PS300-PM; △: 70% PS300-PM; ×: 90% PS300-PM. (**b**) EVMs: ●: IBU crystals; ○: 10% PS300-EVM; ◇: 30% PS300-EVM; □: 50% PS300-EVM; △: 70% PS300-EVM; ×: 90% PS300-EVM. (**c**) SHMs: ●: IBU crystals; ○: 10% PS300-SHM; ◇: 30% PS300-SHM; □: 50% PS300-SHM; △: 70% PS300-SHM; ×: 90% PS300-SHM.

Figure 8 shows the release profiles of IBU from various samples. In all PMs, the dissolution ratio of IBU increased in comparison with IBU crystals. The EVMs and SHMs containing less than 70% IBU showed a fast dissolution rate and high dissolution ratios compared with the PMs. As determined from SEM images, the pore diameter of PS300 is small (Figure 1a); therefore, IBU may not be reachable in the deep portion of the PS300′s pores in a PM. At the same time, from the results of FT-IR in PMs, IBU calcium salt seems to form at the surface. The samples prepared using the SH method showed a high dissolution rate of IBU compared to the EV method. The EV method seems to show that IBU could reach into a deep part of the PS300 when the ethanol was evaporated. On the PS300′s surface, the IBU might be multilayered, and a region of IBU recrystallized. That is, excessive amounts of IBU can form crystals on the surfaces of the PS300; therefore, IBU crystals seemed to become the rate-limiting factor, and the dissolution rate was delayed.

On the other hand, fast release of IBU suggested that adsorption of IBU ended before it reached into the deeper parts of the PS300; that is, sublimed IBU appears to reach into only the shallow regions of the PS300. These behaviors were the same in FLR, which we have previously reported [37].

## 4. Conclusions

In this study, IBU crystals were adsorbed via EV and SH with PS300. IBU crystals were amorphized by adsorbing onto PS300 at adequate mixing ratios. At the same time, IBU calcium was also formed on the surface of the PS300. From the FTIR results, a peak shift of the C=O stretching vibration band to a higher frequency due to the IBU molecules was observed, suggesting an intermolecular interaction between the IBU and PS300. An improvement in the dissolution rate was observed for both the EV and SH methods. Additionally, the petal-like structure of PS300 was found to be involved in the amorphous state and calcium salt of IBU, suggesting that it was essential for enhancing IBU solubility.

The adsorption of drugs to the porous calcium silicate, PS300, in this study, did not require an organic solvent for the SH method. Our findings indicated that this is a novel method for improving the solubility of poorly water-soluble drugs.

PS300 has a spherical shape and good flowability. When PS300 is used in the dosage-form design of poorly water-soluble drugs, it may represent an easy-to-use additive for solid dosage forms, such as tablets. We did not, however, consider the pharmacokinetics of adsorbed and released IBU from PS300. In future research, we should consider changing the pharmacokinetics parameters of IBU, whose solubility improved with PS300 when it was applied in solid dosage forms.

## Figures and Tables

**Table 1:** Composition of various samples.

Content of IBU					
wt/wt%	10	30	50	70	90
**Weight Mixing Ratio**					
PS300	9	7	1	3	1
IBU	1	3	1	7	9

**Table 2:** The characteristics of PS300 and CaSiO_3_.

Carrier	Average Particle Diameter (μm)	Angle of Repose (°)	Density (g/cm^3^)	Specific Surface Area (m^2^/g)	Total Pore Volume (cm^3^/g)
PS300	280.20	18.0	2.6085	177.20	0.723
CaSiO_3_	14.82	47.9	2.5810	1.33	0.003
